# Vulnerability of Triple-Negative Breast Cancer to Saponin Formosanin C-Induced Ferroptosis

**DOI:** 10.3390/antiox11020298

**Published:** 2022-01-31

**Authors:** Hsin-Chih Chen, Han-Hsuan Tang, Wei-Hsiang Hsu, Shan-Ying Wu, Wen-Hsing Cheng, Bao-Yuan Wang, Chun-Li Su

**Affiliations:** 1 Department of Human Development and Family Studies, National Taiwan Normal University, Taipei 106, Taiwan; 60506011e@ntnu.edu.tw; 2 Graduate Program of Nutrition Science, School of Life Science, National Taiwan Normal University, Taipei 116, Taiwan; d09b48005@ntu.edu.tw (H.-H.T.); d49818002@gm.ym.edu.tw (W.-H.H.); shanyingwu@tmu.edu.tw (S.-Y.W.); 61051005s@gapps.ntnu.edu.tw (B.-Y.W.); 3 Genome and Systems Biology Degree Program, Academia Sinica and National Taiwan University, Taipei 106, Taiwan; 4 Department of Microbiology and Immunology, School of Medicine, College of Medicine, Taipei Medical University, Taipei 110, Taiwan; 5 Department of Food Science, Nutrition, and Health Promotion, Mississippi State University, Starkville, MS 39762, USA; wcheng@fsnhp.msstate.edu

**Keywords:** formosanin C, breast cancer, ferroptosis potential index, ferritinophagy, gene database

## Abstract

Targeting ferritin via autophagy (ferritinophagy) to induce ferroptosis, an iron- and reactive oxygen species (ROS)-dependent cell death, provides novel strategies for cancer therapy. Using a ferroptosis-specific inhibitor and iron chelator, the vulnerability of triple-negative breast cancer (TNBC) MDA-MB-231 cells to ferroptosis was identified and compared to that of luminal A MCF-7 cells. Saponin formosanin C (FC) was revealed as a potent ferroptosis inducer characterized by superior induction in cytosolic and lipid ROS formation as well as GPX4 depletion in MDA-MB-231 cells. The FC-induced ferroptosis was paralleled by downregulation of ferroportin and xCT expressions. Immunoprecipitation and electron microscopy demonstrated the involvement of ferritinophagy in FC-treated MDA-MB-231 cells. The association of FC with ferroptosis was strengthened by the results that observed an enriched pathway with differentially expressed genes from FC-treated cells. FC sensitized cisplatin-induced ferroptosis in MDA-MB-231 cells. Through integrated analysis of differentially expressed genes and pathways using the METABRIC patients’ database, we confirmed that autophagy and ferroptosis were discrepant between TNBC and luminal A and that TNBC was hypersensitive to ferroptosis. Our data suggest a therapeutic strategy by ferroptosis against TNBC, an aggressive subtype with a poor prognosis.

## 1. Introduction

According to the World Health Organization, breast cancer is the most prevalent cancer and ranked 5th among cancer-related deaths in 2020. In light of its high heterogeneity, breast cancer is clinically divided into three main subtypes based on the expression of hormone receptors, namely estrogen receptor (ER) and progesterone receptor (PR), and human epidermal growth factor receptor 2 (HER2): luminal, HER2-positive, and triple-negative breast cancer (TNBC). The luminal subtype expresses ER, PR, or both, and is further subdivided into luminal A (HER2-negative) and B (HER2-positive) groups [1]. Luminal A, luminal B, HER2-positive, and TNBC cases account for up to 70%, 10–20%, 5–15%, and ~15% of all breast cancer patients, respectively [2,3]. Compared to luminal A, luminal B has a worse prognosis [4]. Drug targeting of HER2 is known to substantially improve the prognosis of HER2-positive breast cancer [5]. TNBC is generally more aggressive, highly heterogeneous, difficult to treat, and frequently present at distant metastases, leading to a poor prognosis and a high relapse rate, mainly due to the lack of targeted therapies and the development of resistance mechanisms at the early stages of the carcinogenesis [3].

Proposed in 2012, ferroptosis is a new form of cell death that is dependent on intracellular ferrous iron (Fe^2+^) and free radical lipid peroxidation [6]. Enhancement of ferroptosis can be initiated via a form of cargo-specific autophagy (ferritinophagy), in which iron storage protein ferritin is degraded [7]. Ferroptosis is morphologically, biochemically, and genetically different from apoptosis, necrosis, and autophagy [6]. Induction of ferroptosis is initially described by the small molecule erastin, which suppresses system Xc^−^, a cystine-glutamate antiporter that provides adequate concentrations of cystine for the synthesis of glutathione [8]. Acyl-CoA synthetase long-chain family member 4 preferably catalyzes the esterification of CoA to long-chain polyunsaturated fatty acids and activates the corresponding fatty acids for phospholipid biosynthesis or fatty acid oxidation, and thus plays an essential and regulatory role in ferroptosis [9]. Recently, erastin and the anti-cancer drug sorafenib have been shown to selectively eliminate various forms of cancer cells through induction of ferroptosis [10,11,12,13,14,15,16,17,18]. Likewise, erastin and piperazine erastin are known to suppress tumor growth in animals [13,17]. The efficacy of erastin chemotherapy is improved in combination with certain chemotherapeutic drugs, such as temozolomide, cisplatin, cytarabine/ara-C, and doxorubicin/Adriamycin [19,20]. Although the roles of ferroptosis in tumor occurrence, progression, and treatment warrant further investigation, current evidence suggests that ferroptosis inducers are candidates for the treatment of ferroptosis-susceptible tumors [21,22]. Activation of such alternative cell death pathways may overcome drug resistance from existing chemotherapeutics, and could therefore be possible new drug targets.

Although agents that trigger ferroptosis may have unique clinical applications to target cancer cells highly resistant to apoptosis, factors that influence ferroptosis are complex and not fully understood. These factors include transporters and enzymes that regulate redox homeostasis and metabolism of iron, amino acids, and lipids [23]. Currently, treatment of patients with TNBC relies mainly on systemic chemotherapy [24]. In the present study, formosanin C (FC), a diosgenin saponin, was found to trigger ferroptosis and increase chemosensitivity to cisplatin in TNBC MDA-MB-231 cells. Gene analyses using a database of patients with breast cancers revealed a disparity in sensitivity to ferroptosis between luminal A and TNBC subtypes. We utilized the concept of precision medicine to characterize and counteract molecular aberrations of TNBC in the context of targeted drugs, rather than the use of systemic chemotherapy alone, and identified ferroptosis as a targetable metabolic niche in TNBC.

## 2. Materials and Methods

### 2.1. Reagents

All chemicals were obtained from Sigma (St. Louis, MO, USA) unless otherwise indicated. The compounds were: a ferroptosis inducer, 1S,3R-Ras-selective lethal small molecule 3 (RSL3, Selleck Chemicals, Houston, TX, USA); a breast cancer targeted therapeutic, lapatinib (Tykerb^®^; GlaxoSmithKline plc., Brentford, Middlesex, UK); a novel, pure (>98.9% purity), and structurally defined FC (a gift from Dr. Shen-Jeu Won, College of Medicine, National Cheng Kung University, Tainan, Taiwan) [25]; garcinielliptone FC [26] and justicidin A [27], gifts from Dr. Chun-Nan Lin (School of Pharmacy, Kaohsiung Medical University, Kaohsiung, Taiwan); dyes of 2′,7′-dichlorodihydrofluorescein diacetate (H_2_DCFDA), C11-BODIPY, and Phen green™ SK diacetate (Thermo Fisher Scientific Inc., Waltham, MA, USA).

For Western blot analysis, rabbit polyclonal anti-microtubule-associated proteins 1A/1B light chain 3B (LC3B, ab48394), and anti-SLC40A1 (ferroportin, ab58695) antibodies were obtained from Abcam (Cambridge Science Park, Cambridge, UK). Rabbit monoclonal anti-ferritin heavy chain 1 (FTH1, 4393), anti-PARP (9532), and anti-xCT/SLC7A11 (12691) antibodies and rabbit polyclonal anti-caspase 3 (9662) antibody were obtained from Cell Signaling Technology (Danvers, MA, USA). Other antibodies against various proteins were purchased from the following vendors: mouse monoclonal anti-β-actin antibody (Sigma, A3854); goat anti-rabbit (H + L, Millipore Corp., Billerica, MA, USA, AP307P), and goat anti-mouse IgM + IgG + IgA (H + L, Millipore Corp., AP124P) horseradish peroxidase conjugate antibodies.

### 2.2. Cell Culture

Human breast cancer MCF-7 and MDA-MB-231 cell lines from American Type Culture Collection (Rockville, MD, USA) were maintained in Dulbecco’s modified Eagle medium (GIBCO BRL, Gaithersburg, MD, USA) and supplemented with penicillin and streptomycin as well as 10% fetal bovine serum (GIBCO BRL) in an incubator with humidified atmosphere and 5% carbon dioxide at 37 °C.

### 2.3. Cell Population Growth Determination

The sulforhodamine B assay was performed to determine cell density based on the cellular protein content [28]. Briefly, cells were stained with 0.1% (*w/v*) sulforhodamine B dissolved in 1% acetic acid (Baker, J.T.; Center Valley, PA, USA), and the protein-bound dye was extracted with a Tris buffer (20 mM; pH = 10; Bionovas Biotechnology Co., Ltd., Toronto, ON, Canada). The absorbance of each well was measured at 540 nm using a microplate reader (Synergy HT, BioTek, Winooski, VT, USA).

### 2.4. Flow Cytometry

Cells were stained with H_2_DCFDA (100 µM), C11-BODIPY (10 µM), and Phen green SK (10 µM) in the dark at room temperature for 30 min to determine levels of intracellular ROS formation [29], lipid peroxidation [30], and ferrous iron [31], respectively, and 10,000 gated cells of each condition were subsequently analyzed by a flow cytometer (LSRFortessa, Becton Dickinson, Lexington, KY, USA).

### 2.5. Determination of Glutathione Peroxidase 4 (GPX4) Levels

Whole cell lysates (100 µg) were prepared for analyses of GPX4 levels using an ELISA kit for GPX4 (Cloud-Clone Corp., Katy, TX, USA) according to the manufacturer’s protocol. The absorbance was measured at 450 nm using a microplate reader (Synergy HT, BioTek, Winooski, VT, USA).

### 2.6. Western Blot Analysis

Whole cell extracts were prepared with the use of the M-PER lysis buffer, as previously described [32]. The proteins (30 µg) were then loaded onto 8–12% polyacrylamide gels and separated by SDS-PAGE. After being transferred to polyvinylidene fluoride membranes (Perkin Elmer, Santa Clara, CA, USA), the membranes were blocked with 5% skim milk in Tris buffer saline with Tween 20 (20 mM Tris, 150 mM NaCl, 0.1% Tween, pH = 7.4), incubated with primary and secondary antibodies (0.1 µg/mL), and then with chemiluminescent horseradish peroxidase substrate (Millipore Corp.). The protein signals were detected using the Biospectrum Imaging System (Universal Hood II, Bio-Rad Laboratories, Hercules, CA, USA) and analyzed by ImageJ 1.51j8 (National Institutes of Health, Bethesda, MD, USA).

### 2.7. Transmission Electron Microscopy

Cells were incubated with propylene oxide and then exposed to propylene oxide/Epikote. The blocks embedded with the Epon-Araldite mixture (Electron Microscopy Sciences, Hatfield, PA, USA) were sectioned for imaging under a transmission electron microscope (HITACHI-7000, Hitachi, Tokyo, Japan). For immunogold labeling, the ultrathin sections of the cells on the nickel grids were blocked with the SuperBlock^TM^ blocking buffer (Thermo Fisher Scientific Inc.), followed by incubation with mouse monoclonal anti-FTH1 (1:100, Santa Cruz Biotechnology, Inc., Dallas, TX, USA, sc-376594) and rabbit polyclonal anti-nuclear receptor co-activator 4 (NCOA4, 1:100, Abcam, ab222071) antibodies. The grids incubated with gold-containing goat polyclonal anti-mouse (20 nm; 1:10, Abcam, ab27242) and anti-rabbit (12 nm; 1:10, Abcam, ab105298) secondary antibodies were stained with saturated uranyl acetate and lead citrate, respectively. The images were examined under a transmission electron microscope (HITACHI-7000, Hitachi, Tokyo, Japan).

### 2.8. Immunoprecipitation

Immunoprecipitation was performed with the Protein A/G Magnetic Beads system (Thermo Fisher Scientific Inc.) according to the manufacturer’s protocol. Briefly, anti-LC3 antibody (5 μg/mL, Medical & Biological Laboratories Co., Ltd., Nagoya, Japan, M152-3) was coupled to protein A/G plus magnetic beads by incubation at 4 °C for 3–4 h. Cells were lysed with a lysis buffer (Sigma, C3228), and protein (100–150 μg) from the lysates were incubated with the anti-LC3 antibody in the presence of magnetic beads at 4 °C overnight. The beads were then collected with a DynaMag™-2 Magnet (Thermo Fisher Scientific Inc.). After removing the supernatant, the immunoprecipitates were washed three times with the lysis buffer, and the proteins (20 µg) were subjected to SDS-PAGE for Western blot analysis.

### 2.9. RNA Sequencing

Total RNAs were isolated from MDA-MB-231 cells using the Quick-RNA™ Miniprep Plus Kit (Zymo Research, Irvine, CA, USA). The RNAs were subjected to the SimpliNano™-Biochrom Spectrophotometer (Biochrom, Holliston, MA, USA) for purity and quantity and the Qsep 100 DNA/RNA Analyzer (BiOptic Inc., New Taipei City, Taiwan) for integrity, respectively. RNA fragmentation and library preparation (KAPA Biosystems, Roche, Basel, Switzerland) were carried out by the NovaSeq 6000 System (Illumina, San Diego, CA, USA), through which the constructed libraries were analyzed by 150 bp paired-end high-throughput sequencing at Biotools Co., Ltd. (New Taipei City, Taiwan). Briefly, read pairs mapping from each sample were aligned to the reference genome (i.e., *H. sapiens*, GRCh38) by the HISAT2 software (v2.1.0) and the results were reported following the “fragments per kilobase of transcript per million mapped reads” method, known as FPKM, which quantile normalized all samples. Moreover, for gene expression analysis, the “Trimmed Mean of M-values” normalization was performed using DEGseq (v1.36.1) [33] without biological duplicate, and the “Relative Log Expression” normalization was performed using DESeq2 (v1.22.1) [34,35] with biological duplicate. DEGs analysis of these two conditions was performed in R using DEGseq (without biological replicate) and DESeq2 (with biological replicate), which is based on negative binomial distribution and Poisson distribution model, respectively [36,37,38]. The resulting *p*-values were adjusted using the Benjamini and Hochberg’s approach for controlling the false discovery rate (FDR). DEGs were defined as genes with *p*-value < 0.05.

### 2.10. METABRIC

Transcriptomic data from studies of the METABRIC [39,40] which is publicly accessible and de-identified were analyzed via the cBioPortal (https://www.cbioportal.org/, accessed on 28 April 2021) on 28 April 2021 [41,42]. Microarray transcriptomes (*n* = 1904) of breast cancer patients were divided into luminal A (*n* = 1369), luminal B (*n* = 109), TNBC (*n* = 299), and HER2-enriched (ER-negative, PR-negative, and HER2-positive, *n* = 127) according to the ER, PR, and HER2 status. The Nottingham prognostic index, overall survival status, normalized gene expression data, and the DEGs with a FDR at <0.05 between patients with luminal A and TNBC were downloaded for further analysis. As for microarray transcriptome, the RNA isolation and hybridization and data preprocessing were described thoroughly in the original METABRIC paper [39]. Briefly, the RNA was isolated, amplified, and hybridized onto Illumina Human HT-12 v3 Expression Beadchips, and then scanned on the Illumina BeadArray Reader. The raw data were preprocessed by spatial artifact correction, summarization, and normalization to obtain the log 2 intensity [39]. *p*-values and FDR were analyzed by the Student’s *t*-test and the Benjamini-Hochberg procedure, respectively. Finally, the DEGs between luminal A and TNBC subtypes were defined as genes with FDR < 0.05.

### 2.11. Over-Represented Pathway Analysis and Gene Set Enrichment Analysis (GSEA)

The DEGs were applied to the Consensus Path Database (CPDB) v34 (http://cpdb.molgen.mpg.de/; accessed date: 28 April 2021) [43] for query of over-represented pathways (minimum overlap with input list, 2; *p*-value cutoff, 0.01) collected in the Wikipathways [44]. GSEA [45], a computational method that evaluates whether an a priori defined set of genes exhibits statistically concordant significance between two biological states, was performed using GSEA v4.1.0 software with 1000 permutations. “Ferroptosis”, “Fatty Acid Biosynthesis”, “Lipid Metabolism Pathways”, “Senescence and Autophagy in Cancer”, and “Focal Adhesion-PI3K-Akt-mTOR-signaling pathway” gene sets deposited in the Wikipathways [44] were downloaded from the Molecular Signature Database v7.4 (https://www.gsea-msigdb.org/gsea/msigdb/; accessed date: 28 April 2021). “Difference of class means” was adopted for ranking of genes in the list. Genes whose expression did not differ between groups (log2 fold change = 0) were eliminated from further analysis.

### 2.12. Prognostic Ferroptosis DEGs

The experimentally validated ferroptosis driver (promoting ferroptosis) and suppressor genes (inhibiting ferroptosis) were downloaded from the FerrDb v1 [46] (http://www.zhounan.org/ferrdb/; accessed date: 24 November 2021). FerrDb collected manually annotated and experimentally validated ferroptosis modulators from papers in PubMed. To obtain the DEGs involved in ferroptosis modulation (ferroptosis DEGs), the DEGs we obtained from the METABRIC analysis were intersected with both the ferroptosis drivers and suppressors using VENNY 2.0 [47] (https://bioinfogp.cnb.csic.es/tools/venny/; accessed date: 24 November 2021). Genes that overlapped between drivers and suppressors were excluded to avoid ambiguity. In order to gain further insight into the roles of ferroptosis genes in the prognosis of the breast cancer patients, the overall survival status and expression of the ferroptosis DEGs from the METABRIC were applied to the Kaplan–Meier plotter (https://kmplot.com/analysis/; accessed date: 24 November 2021) for univariate Cox regression analysis and patients were split by the “Auto select best cutoff” option. Ferroptosis DEGs with a *p*-value and FDR of the Hazard ratio <0.0001 and ≤0.05, respectively, were selected as the prognostic ferroptosis DEGs.

### 2.13. Ferroptosis Potential Index (FPI)

To quantify the propensity for ferroptosis in luminal A and TNBC patients, FPI [48] was calculated. Briefly, the index was established based on the expression of the 20 prognostic ferroptosis DEGs, including 10 driver genes (*GOT1*, *G6PD*, *HILPDA*, *SLC1A5*, *LONP1*, *TF*, *LINC00472*, *NCOA4*, *FLT3*, and *CDO1*) and 10 suppressor genes (*FANCD2*, *GCH1*, *HSF1*, *ENPP2*, *NQO1*, *SCD*, *SQSTM1*, *ISCU*, *SLC40A1*, and *TP63*). The sample-wise enrichment scores of the driver and suppressor gene sets were calculated and normalized using gene set variation analysis (GSVA), a method of GSEA for characterizing pathways or signature summaries from a gene expression dataset (i.e., the expression of the patients with TNBC and luminal A), in the “GSVA” R package [49]. The enrichment score of the driver gene set minus that of the suppressor gene set was defined as FPI. The larger the FPI is, the higher the potential for ferroptosis induction is.

### 2.14. Statistical Analysis

All data were analyzed with the use of at least three independent experiments, and were presented as means ± standard errors of the means (SEMs). Student’s *t*-test and one-way ANOVA were performed for statistical analysis (Statistical Package for the Social Science 22.0 and 28.0 for Windows). The level of significance was set as α = 0.05.

## 3. Results

### 3.1. The TNBC MDA-MB-231 Cells Are More Sensitive to Ferroptosis Than the Luminal A MCF-7 Cells, and FC Is Identified as a Potent Ferroptosis Inducer

**Figure 1:**
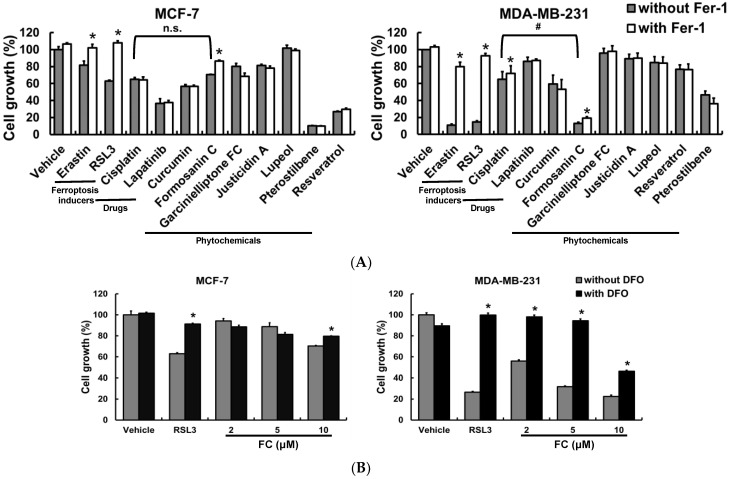

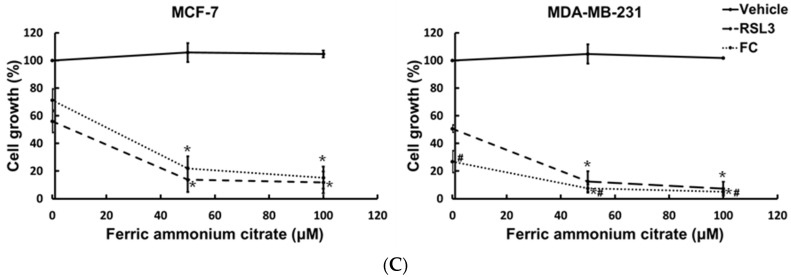
FC-induced ferroptosis is more effective in MDA-MB-231 cells. (**A**) Effect of various types of compounds on the growth of MCF-7 and MDA-MB-231 cells. Cells received treatments of erastin (10 μM), RSL3 (5 μM), cisplatin (10 μM), lapatinib (0.5 μM), curcumin (20 μM), formosanin C (FC; 10 μM), garcinielliptone FC (20 μM), justicidin A (10 μM), lupeol (100 μM), pterostilbene (100 μM), and resveratrol (100 μM) separately in the presence and absence of ferroptosis inhibitor ferrostatin-1 (Fer-1; 5 μM) for 24 h. Cell growth were analyzed by sulforhodamine B assay. * Compared to without Fer-1, *p* < 0.05; Student’s *t*-test. # *p* < 0.05; Student’s *t*-test. n.s., not significant. (**B**) Effect of iron chelator on the growth of the cells received FC. Cells received treatments of FC in the presence and absence of deferoxamine (100 μM) for 24 h. The ferroptosis inducer, RSL3 (5 μM), was used as a positive control. * Compared to without deferoxamine, *p* < 0.05; Student’s *t*-test. DFO denotes deferoxamine. (**C**) Iron-enhanced cell growth inhibition of FC. Cells received treatments of RSL3 (1 μM) or FC (5 μM) in the presence and absence of ferric ammonium citrate for 24 h. * Compared to 0 μM of ferric ammonium citrate, *p* < 0.05; Student’s *t*-test. # Compared to MCF-7 cells under the same experimental conditions, *p* < 0.05; Student’s *t*-test.

Although luminal A is highly prevalent [2], TNBC has a poor prognosis and a high risk of relapse [3]. To determine the sensitivity of these two subtypes to ferroptosis, the TNBC MDA-MB-231 and the luminal A MCF-7 cells were treated with the ferroptosis inducer, erastin (a system Xc^−^ inhibitor [6]) or RSL3 (a GPX4 inhibitor [13]). We found that RSL3 and erastin were efficacious in the suppression of cell growth, and the trend was more pronounced in MDA-MB-231 than in MCF-7 cells (Figure 1A). Likewise, FC demonstrated more potent suppression of growth of MDA-MB-231 cells compared with MCF-7 cells. The inhibitory effects of these three chemicals could be reversed when the cells were incubated with ferrostatin-1 (Fer-1), a ferroptosis inhibitor that quenches Fe^2+^-dependent lipid reactive oxygen species (ROS) [29]. By contrast, co-treatment with Fer-1 did not influence the effect of the US FDA-approved breast cancer drug (lapatinib) [50] or six autophagy-inducing phytochemicals (curcumin, garcinielliptone FC, justicidin A, lupeol, pterostilbene, and resveratrol) in MDA-MB-231 and MCF-7 cells. FC exhibited the strongest cell growth inhibition among all tested phytochemicals and anti-cancer drugs, including cisplatin, in MDA-MB-231.

It is noteworthy that, among the three phytochemicals that inhibited the growth of MDA-MB-231 cells, FC (10 µM) was the only one whose cell growth inhibitory effect could be significantly reversed in the presence of Fer-1. To corroborate this observation, lower concentrations of FC (2 and 5 μM) were employed (Figure 1B). Treatment with FC demonstrated a dose-dependent inhibition (2*–*10 µM), which was attenuated in the presence of deferoxamine (an iron chelator [6]) in MDA-MB-231 cells. FC at 2 and 5 μΜ did not significantly inhibit MCF-7 cells’ growth, and therefore the inhibitory effect of deferoxamine was not possible to be observed in MCF-7 cells. These results collectively suggest that the inhibitory effect of Fer-1 and deferoxamine against FC’s activity demonstrated that FC-triggered iron and lipid ROS-dependent ferroptotic cell death are involved in FC’s mechanism. To determine whether iron can promote FC-induced cell death, an iron source (ferric ammonium citrate) was co-cultured with FC or RSL3. As shown in Figure 1C, treatment with ferric ammonium citrate alone did not change the population growth of either cell line; however, it sensitized the growth of MDA-MB-231 and MCF-7 cells to the inhibitory effect of RSL3 or FC (Figure 1C), suggesting that iron enhanced either FC- or RSL3-induced ferroptosis. It is noteworthy that the FC-reduced cell growth was more effective (*p* < 0.05) in ferric ammonium citrate-treated MDA-MB-231 cells than in MCF-7 cells that had received the same treatment. Altogether, these data suggest that FC was an effective ferroptosis activator, and the efficacy was greater in MDA-MB-231 cells than in MCF-7 cells.

### 3.2. MDA-MB-231 Cells Are More Effective Than MCF-7 Cells to FC-Induced Cytosolic and Lipid ROS Production and GPX4 Depletion

**Figure 2:**
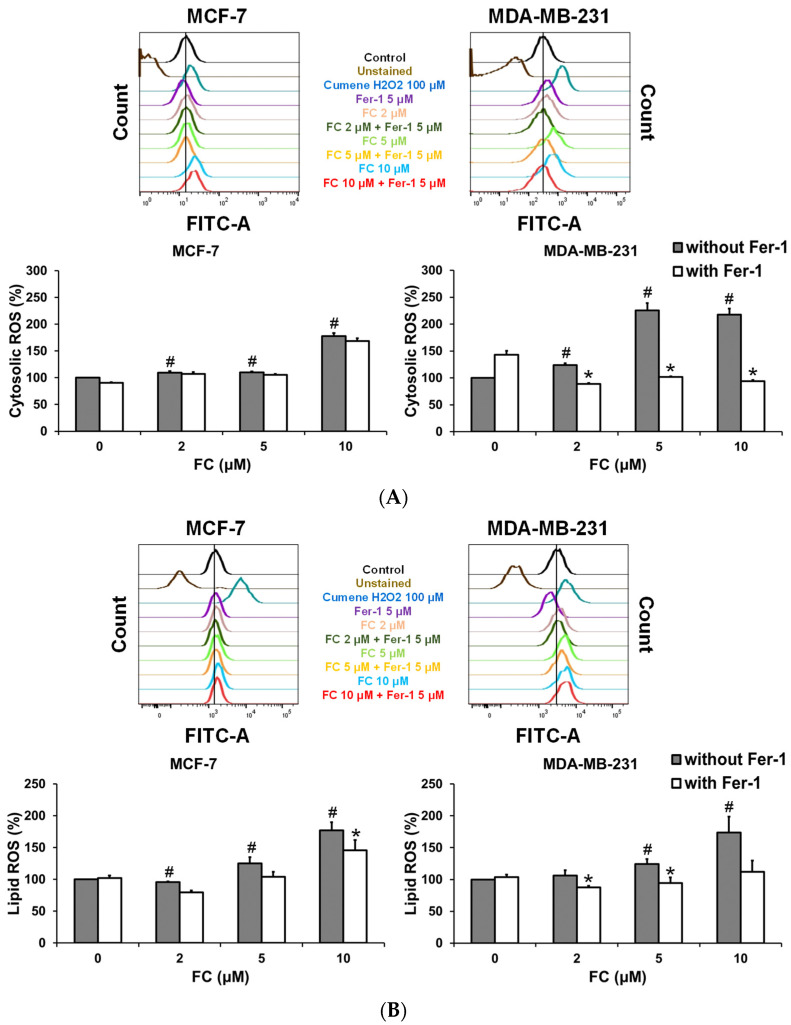

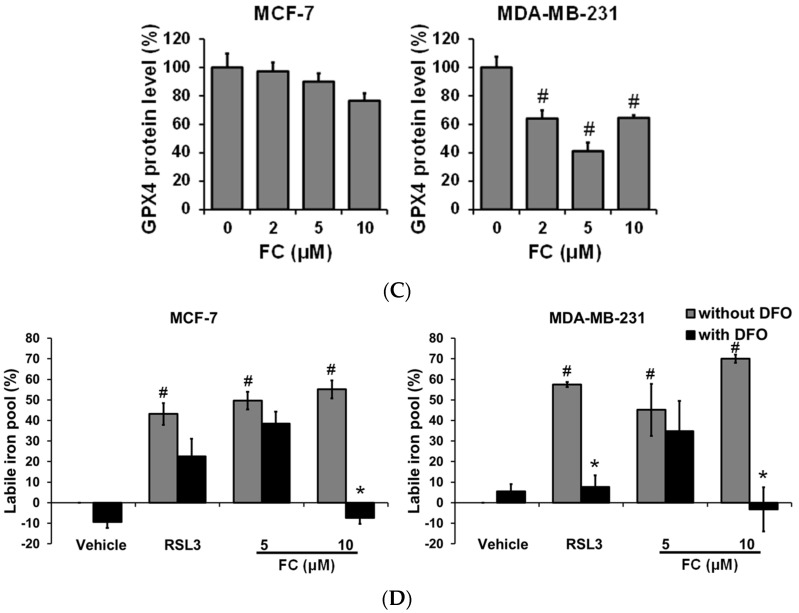
FC increases ROS generation and iron accumulation. (**A**) FC elevated cytosolic ROS. (**B**) FC elevated lipid ROS. Cells received treatments of FC in the presence and absence of ferroptosis inhibitor ferrostatin-1 (Fer-1, 5 μM) for 24 h. Cytosolic and lipid ROS were detected using flow cytometry after staining with H_2_DCFDA and C11-BODIPY, respectively. The higher the intensity (the peak shifts to the right) of H_2_DCFDA or C11-BODIPY fluorescence is, the richer the cytosolic or lipid ROS are, respectively. * Compared to without Fer-1, *p* < 0.05; Student‘s *t*-test. # Compared to the corresponding vehicle, *p* < 0.05; Student’s t-test. (**C**) FC-reduced GPX4 protein level. The GPX4 protein levels were detected with enzyme-linked immunosorbent assay kit for GPX4. Cells received treatments of FC for 24 h. # Compared to the corresponding vehicle, *p* < 0.05; Student’s t-test. (**D**) FC increased intracellular iron accumulation. Cells received treatments of FC in the presence and absence of iron chelator deferoxamine (100 μM) for 24 h. Ferroptosis inducer, RSL3 (5 μM), was used as a positive control. Intracellular iron accumulation was detected using flow cytometry after Phen green SK staining. * Compared to without deferoxamine, *p* < 0.05; Student‘s *t*-test. # Compared to the corresponding vehicle, *p* < 0.05; Student’s t-test. DFO denotes deferoxamine.

Because ferroptosis depends on both iron and lipid ROS [6], we sought to determine whether such responses differ between TNBC MDA-MB-231 and luminal A MCF-7 cells. In line with our recent observations on human hepatocellular carcinoma cells [51], FC was identified as a potent ferroptosis inducer in breast cancer cells (Figure 1). Therefore, FC was used to induce ferroptosis for further experiments. ROS can be generated by various mechanisms. The attenuation in ROS formation by Fer-1 was considered ferroptosis-related, as Fer-1 specifically quenches Fe^2+^-dependent formation of ROS [29]. Treatment with FC elevated (*p* < 0.05) cytosolic ROS formation to a greater extent in MDA-MB-231 cells than in MCF-7 cells, and the induction was completely reversed in the presence of Fer-1 in the former but not in the latter cells (Figure 2A). The formation of lipid ROS [30] was induced (*p* < 0.05) by FC and was returned more closely to the basal level in the presence of Fer-1 in MDA-MB-231 cells than in MCF-7 cells (Figure 2B). It is noteworthy that the concentration of FC at which the lipid ROS formation was significantly attenuated by Fer-1 was lower in MDA-MB-231 cells than in MCF-7 cells. Conversely, treatment with FC reduced (*p* < 0.05) GPX4 protein level in MDA-MB-231 cells, but not in MCF-7 cells (Figure 2C). The observation that FC-induced labile iron pool was decreased by the iron chelator, deferoxamine (Figure 2D), provided further support that FC-induced cell growth inhibition (Figure 1B) was coupled with the elevation of intracellular labile iron. Altogether, the elevation of cytosolic and lipid ROS and labile iron levels contributed to FC-induced cell death in association with the depletion of GPX4.

### 3.3. FC-Induced Ferroptosis Parallels Downregulation of Ferroportin and xCT Expressions and Upregulation of LC3 Expression in TNBC MDA-MB-231 Cells

**Figure 3:**
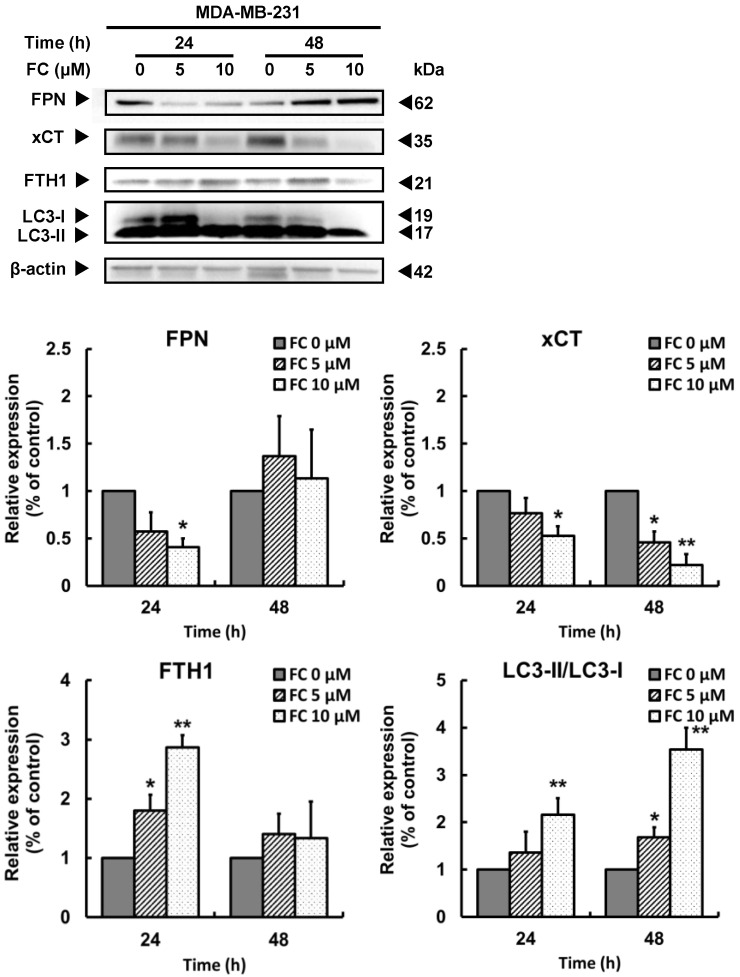
FC changes expressions of proteins related to antioxidant system and iron metabolism. Cells received treatments of FC for 24 and 48 h. Whole cell lysates were prepared and subjected to Western blot analysis using anti-ferroportin, anti-xCT, anti-FTH1, and anti-LC3 antibodies. β-actin antibody was used as an internal control. FPN denotes ferroportin. The intensity of each protein expression band was quantified (*n* = 3). * and ** Compared to the corresponding control (0 μM of FC), *p* < 0.05 and *p* < 0.01, respectively; Student‘s *t*-test.

Because FC-induced downregulation of GPX4 was more effective in the TNBC MDA-MB-231 cells (Figure 2C), proteins associated with anti-oxidation and iron homeostasis were studied. FC treatment of MDA-MB-231 cells resulted in increases (*p* < 0.05) in the protein level of FTH1 at 24 h and LC3-II/LC3-1 at both 24 and 48 h, but resulted in decreases in xCT (SLC7A11, the light chain subunit of system Xc^−^-cystine/glutamate antiporter [8,23]) at both 24 and 48 h and ferroportin at 24 h (Figure 3). In contrast, the FC treatment did not increase FTH1 at 24 h or decrease xCT at 24 h or ferroportin at 24 and 48 h in MCF-7 cells (Figure S1). Altogether, these data suggest that FC-induced ferroptosis in TNBC is associated with suppressed levels of the iron export protein, ferroportin, and the antioxidant capacity protein, xCT.

### 3.4. Confirmation of FC-Induced Ferritinophagy and Ferroptosis in TNBC MDA-MB-231 Cells

**Figure 4:**
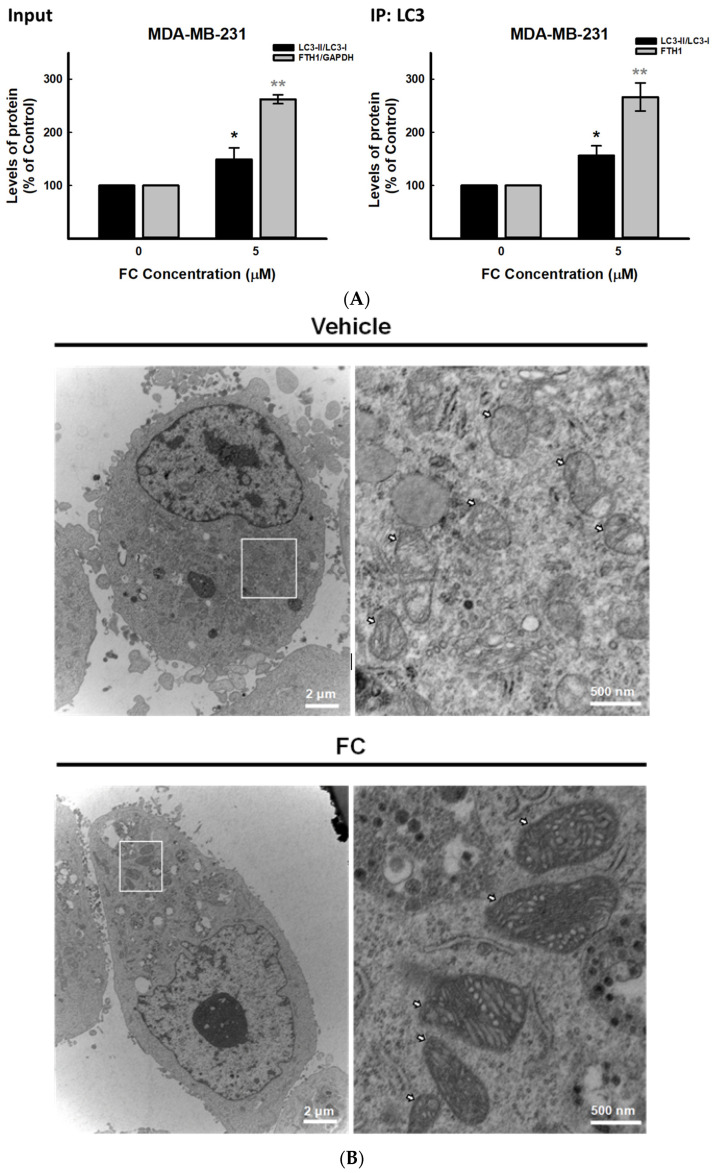

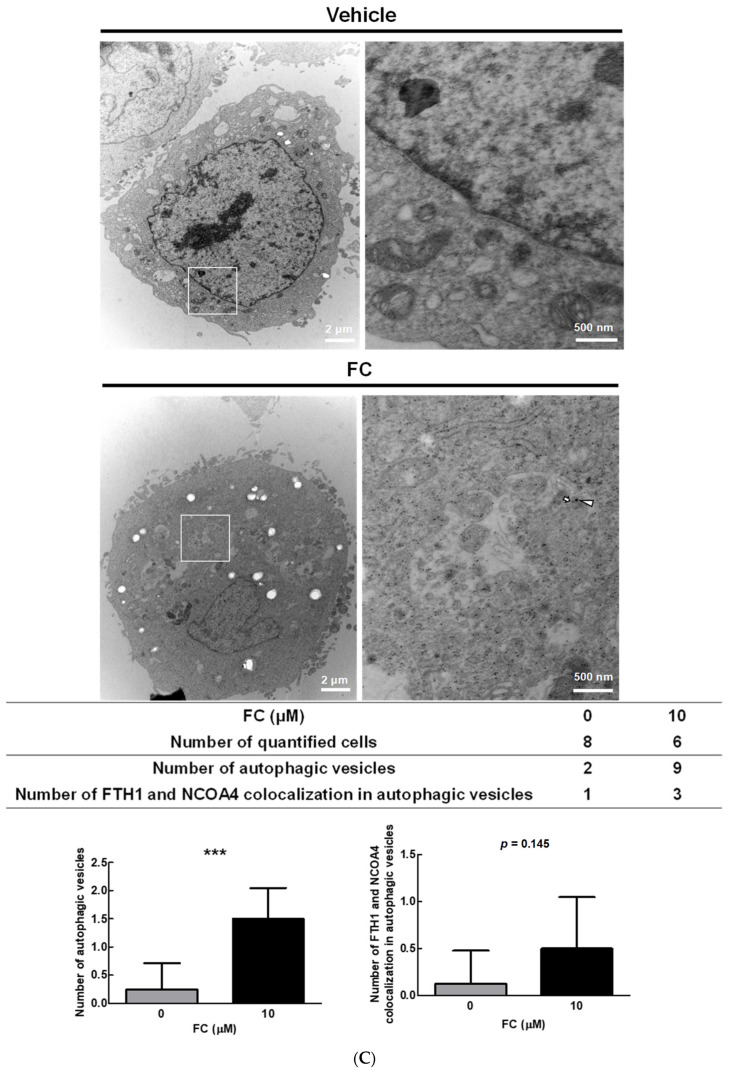
Ferritinophagy in FC-treated MDA-MB-231 cells. (**A**) The interaction of LC3 and FTH1. Cells received treatments of FC for 24 h. The cell lysates were immunoprecipitated using anti-LC3 antibody, and the immune complexes were subjected to Western blot using anti-LC3 and anti-FTH1 antibody, separately. The intensity of each protein expression band was quantified (*n* = 3). * and ** compared to the corresponding control (0 μM of FC), *p* < 0.05 and *p* < 0.01, respectively; Student‘s *t*-test. (**B**) Mitochondrial morphology. The arrow indicates the differences in the morphology of mitochondria between control and FC-treated cells. (**C**) Co-localization of gold-stained ferritin and NCOA4. Cells received treatments of FC (10 μM) for 24 h. Autophagosomes/autolysosomes and gold particles were quantified. *** denote *p* < 0.001; Student’s *t*-test. The arrow and triangle indicate NCOA4 (12 nm) and FTH1 (20 nm), respectively. The images were taken by electron microscopy.

Upregulation of LC3-II/LC3-I, an autophagy marker, (Figure 3) implies that ferritinophagy occurred in FC-treated MDA-MB-231 cells. The analysis of immunoprecipitation demonstrated that FTH1 was co-immunoprecipitated with LC3 in a FC dose-dependent manner (Figure 4A and Figure S2). However, this phenomenon was not observed in the FC-treated MCF-7 cells (Figure S2). Next, the FC-altered ultra-structure of the MDA-MB-231 cells was examined. Transmission electron microscopy analysis revealed that FC caused the distinctive mitochondrial morphology with an increased membrane density (Figure 4B), a reported cytological change in ferroptotic cells [6,14]. The number of autophagic vesicles was significantly increased in the FC treatment (10 µM) compare to the control (*p* = 0.0006). Although the statistical analysis of colocalization between the gold-stained FTH1 and NCOA4 in the autophagic vesicles was not significantly changed in the FC treatment compared to the control (*p* = 0.145), an increasing trend was observed (Figure 4C). These observations confirm the involvement of ferritinophagy in FC-induced ferroptosis in MDA-MB-231 cells. Taken together, our data suggest that induction of ferritinophagy alters cellular labile iron concentration (Figure 2D) and increases the accumulation of cellular lipid ROS (Figure 2B), plausibly leading to ferroptotic cell death (Figure 1B) in MDA-MB-231 cells.

### 3.5. FC Increases Cisplatin Sensitivity of MDA-MB-231 Cells

**Figure 5:**
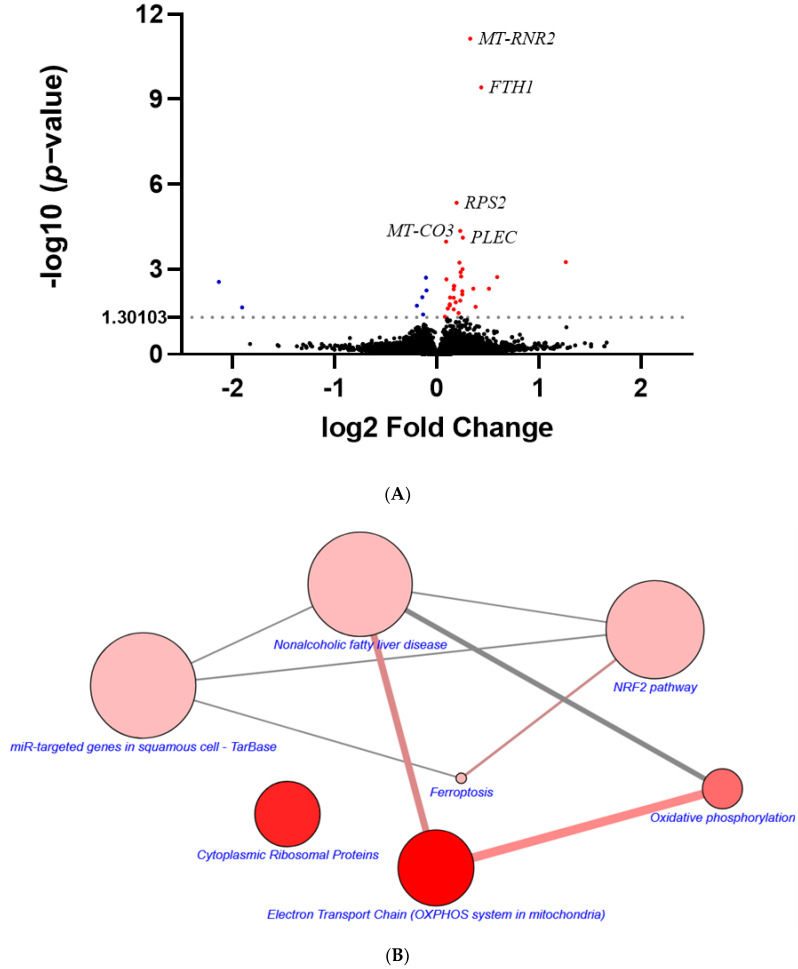
Biological function of FC via bioinformatics analysis. (**A**) FC-altered mRNA expression. Cells received treatments of FC (0.5 μM) for 6 h to avoid secondary responses under a higher treatment concentration for a longer period time. RNA sequencing was carried out to investigate the transcriptomic alteration of FC. Volcano plot displays the log2 fold change (FC/vehicle) and -log10 (*p*-value) of the genes altered by FC. DEGs were defined as genes with *p* < 0.05. Red and blue dots denote upregulated (log2 fold change > 0) DEGs and downregulated (log2 fold change < 0) DEGs, respectively. Black dots denote non-DEGs. The names of the top 5 DEGs are shown. (**B**) The over-represented pathways analyzed using the DEGs between FC treatment and vehicle via CPDB. The size of each dot designates the entity number of genes in the pathway. The intensity of dot color denotes the *p*-value. The darker the color is, the smaller the *p*-value is. The line between two dots was analyzed by the function of these two pathways to show the number of genes overlapping said pathways. The breadth of the line indicates the strength of the correlation between two dots.

**Figure 6:**
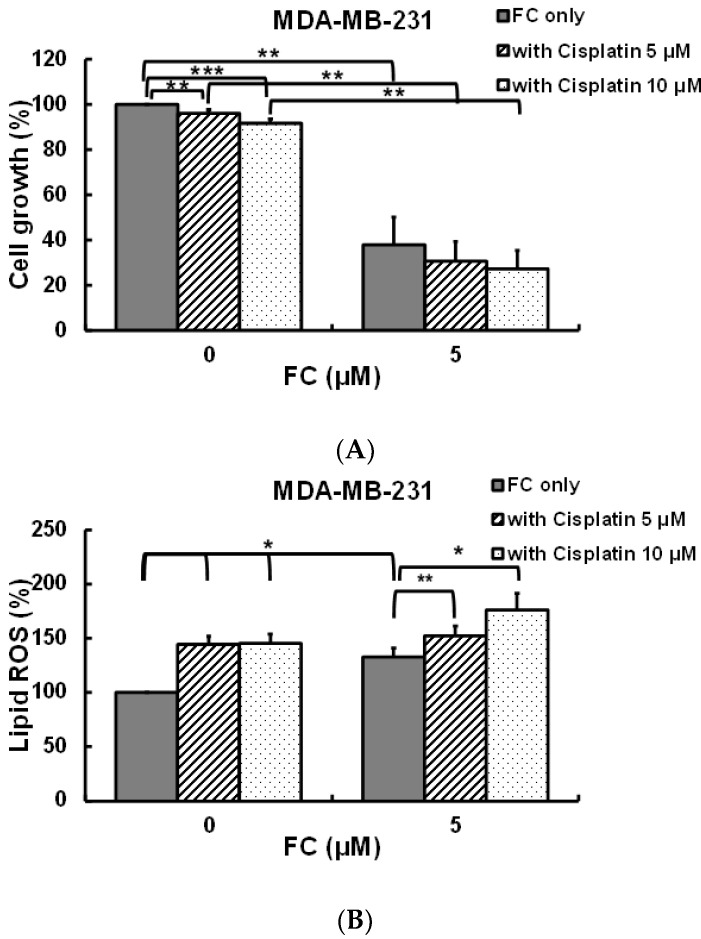
Effect of FC on cisplatin-treated cells. (**A**) Combination of FC and anti-cancer drug cisplatin increased cell growth inhibition. (**B**) Combination of FC and cisplatin increased lipid ROS formation. Cells received treatments of FC in the presence and absence of cisplatin for 24 h. Cell growth was analyzed by sulforhodamine B assay. Lipid ROS were detected using flow cytometry after staining with C11-BODIPY. *, **, and *** denote *p* < 0.05, *p* < 0.01, and *p* < 0.001; Student’s *t*-test.

High throughput RNA sequencing was performed to comprehensively analyze FC-altered genes and pathways using all (n = 38, Table S1) of the DEGs (*p*-value < 0.05) between FC treatment and vehicle (Figure 5A) in TNBC MDA-MB-231 cells. Interestingly, genes involved in iron metabolism (e.g., *FTH1*), electron transport chain (e.g., *MT-CO3*), and the structural constituent of ribosome (e.g., *RPS2*) were differentially expressed (Figure 5A). The DEGs were further queried in the Wikipathways database [44] for over-represented pathways via CPDB [43]. In agreement with the results obtained through biochemical and cellular approaches (Figure 1, Figure 2 and Figure 3), the network diagram (Figure 5B and Table S2) linked “Ferroptosis” as well as its associated biological functions (e.g., “Oxidative phosphorylation” and “NRF2 pathway”) to the action of FC. Ferroptosis has been proven to increase sensitivity to cisplatin in a broad spectrum of cancer cell lines (e.g., non-small cell lung cancer [52], gastric cancer [53], and head and neck cancer [54]). Therefore, the researchers were particularly interested in investigating the ferroptotic and cytotoxic effects of cisplatin in combination with FC in TNBC MDA-MB-231 cells. Co-administration of FC inhibited the growth of the cisplatin-treated MDA-MB-231 cells (Figure 6A). FC treatment at 2 μM significantly enhanced the cell growth inhibition of cisplatin, which was reversed (p < 0.05) in the presence of Fer-1 (Figure S3A), indicating that ferroptotic cell death was involved in the FC-enhanced cell growth inhibition of cisplatin. A low concentration of cisplatin was sufficient to exhibit the cytotoxic effects, suggesting that when used in combination with FC it may be possible to reduce the dose-limiting side effect of cisplatin. Moreover, cisplatin and FC treatment elevated lipid ROS formation in MDA-MB-231 cells (Figure 6B), which was reduced (p < 0.05) in the presence of Fer-1 (Figure S3B). Altogether, these results implicate ferroptosis induction in the sensitization of cisplatin-treated TNBC MDA-MB-231 cells to FC.

### 3.6. Analyses of Data Repositories Strengthen the Observed Discrepancy between TNBC and Luminal A on Ferroptosis and Autophagy as well as Hypersensitivity to Ferroptosis in TNBC

**Figure 7:**
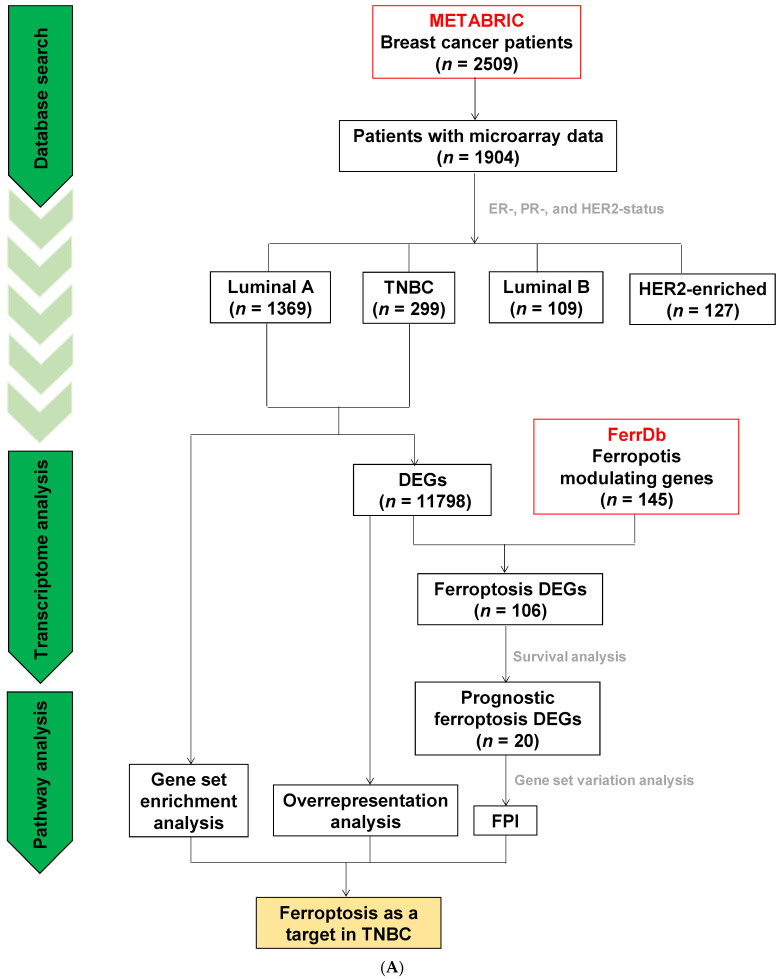

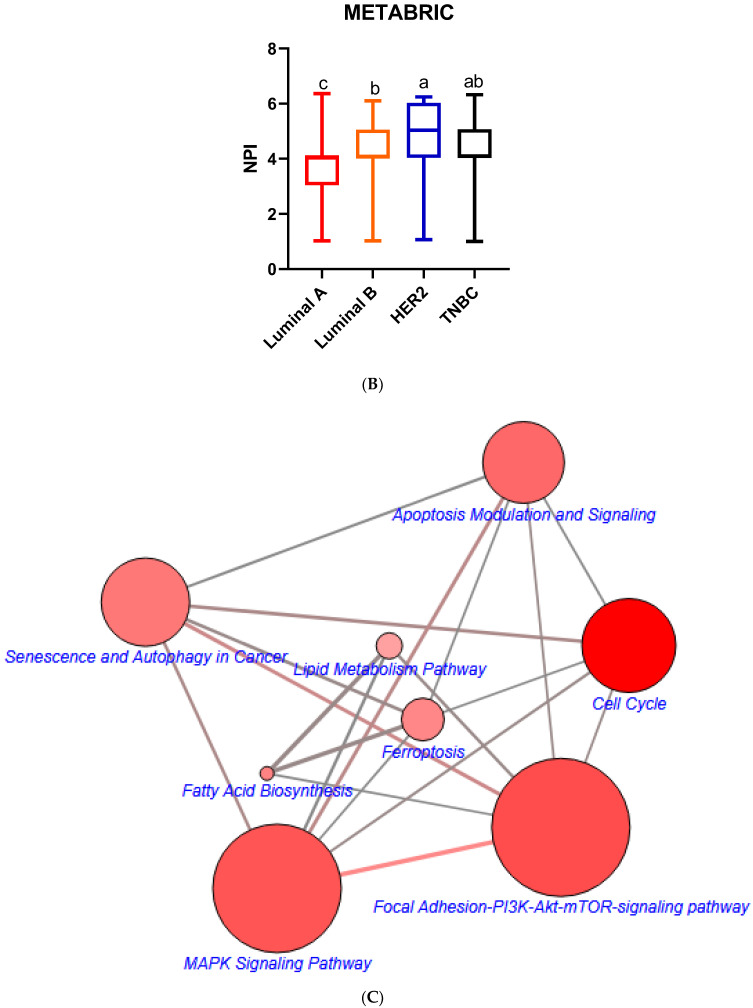

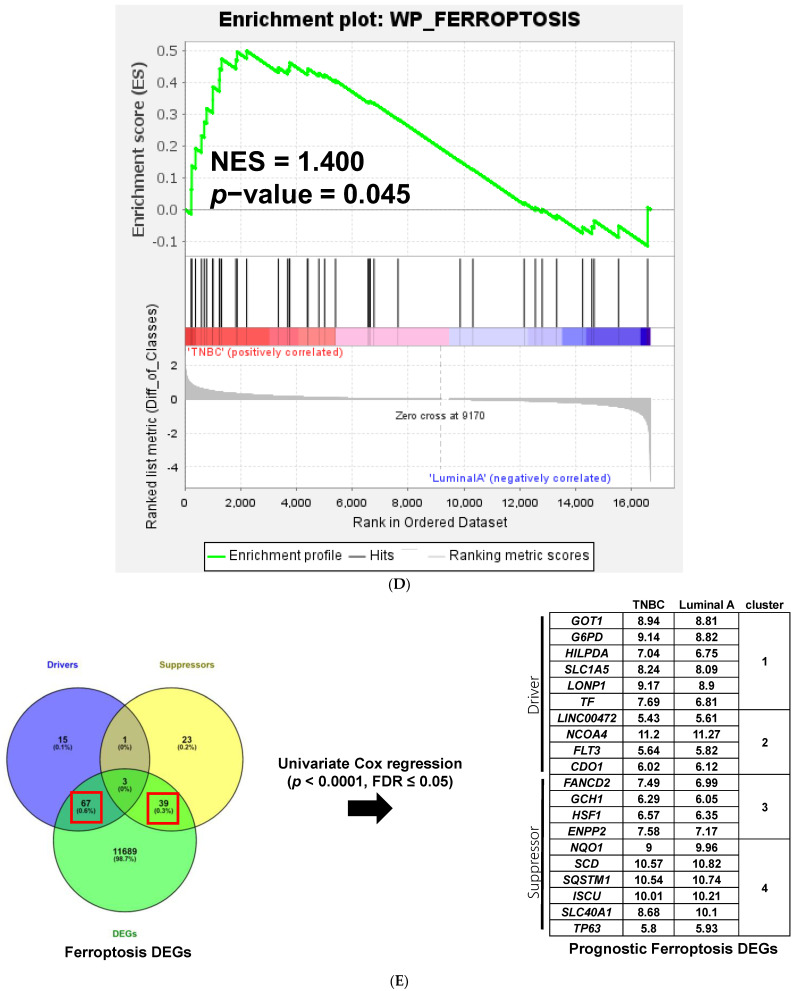

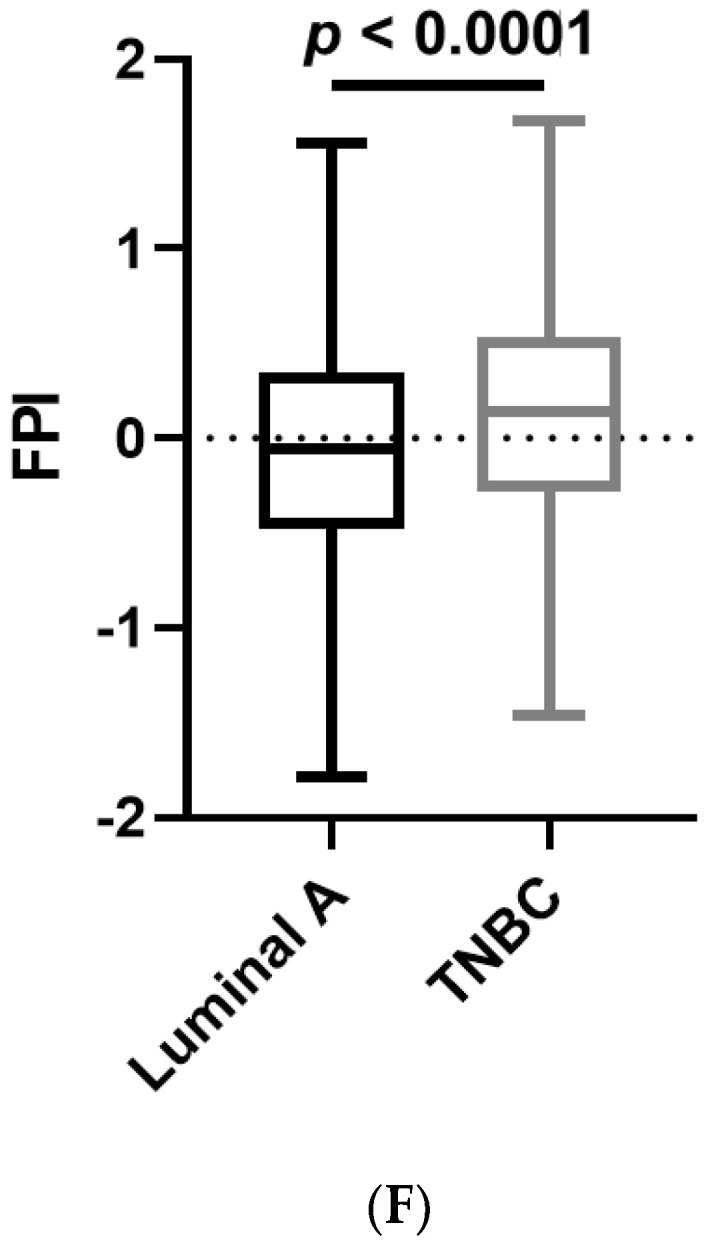
Ferroptosis is shown to account for discrepancy between TNBC and luminal A. (**A**) Flow chart of in silico data collection and analyses. (**B**) Nottingham prognostic index obtained from METABRIC cohort. Differences among groups were analyzed by one-way ANOVA and Tukey’s multiple comparison test. Medians with different subscript letters are significantly different, at *p* < 0.05. (**C**) The over-represented pathways analyzed using the DEGs between patients with TNBC and luminal A via CPDB. The size of each dot designates the entity number of genes in the pathway. The intensity of dot color denotes the *p*-value. The darker the color is, the smaller the *p*-value is. The line between two dots was analyzed by the function of these two pathways to show the number of genes overlapping said pathways. The breadth of the line indicates the strength of the correlation between two dots. (**D**) Identification of ferroptosis as a potential regulator on TNBC using GSEA. The enrichment score is normalized to account for the size of the gene set, demonstrating significant enrichment (*p* < 0.05). NES denotes normalized enrichment score. (**E**) Genes associated with ferroptosis and prognosis of patients with TNBC and luminal A. DEGs (FDR < 0.05) between patients with TNBC and luminal A in the METABRIC cohort were intersected with experimentally validated ferroptosis driver and suppressor genes. The common genes (red boxes) were selected for survival analyses (left panel). Ferroptosis-related prognostic DEGs were shown and the values represented mean of the log2 gene expression (right panel). (**F**) The FPI between patients with TNBC and luminal A. Sample-wise enrichment scores of the prognostic ferroptosis driver DEG set and suppressor DEG set were independently generated using GSVA algorithm. The enrichment score of the prognostic ferroptosis driver DEG set minus that of the prognostic ferroptosis suppressor DEG set was defined as FPI. The horizontal dashed line indicates FPI = 0, which means the potential of ferroptosis is neutral. The lower and upper extents of the box represent the 25th and 75th percentiles, respectively. The parallel line in the box represents the median. The lower and upper extreme of the whisker represents minimum and maximum, respectively (**B**) and (**F**). NPI denotes Nottingham prognostic index.

Figure 7A illustrates the flow chart of the in silico data collection and analyses. The Nottingham prognostic index is a surrogate marker of the aggressiveness of breast cancers and is scored by the size of the tumor, number of lymph nodes involved, and tumor grade. It is a valuable prognostication tool to provide gross projection of survival. Data analyses of all subtypes stored in the METABRIC study [39,40] revealed that the patients with TNBC had a significantly higher Nottingham prognostic index compared to patients with luminal A but not other subtypes (Figure 7B), suggesting that TNBC is more aggressive than the luminal A subtype. To characterize these observations, the DEGs (FDR < 0.05) between patients with TNBC and luminal A from the METABRIC study were used to query the Wikipathways database [44] for over-represented pathways via the CPDB [43]. Several signaling pathways and cell death-related modalities were over-represented between TNBC and luminal A subtypes (Table S3). In the network diagram, “Ferroptosis” and its associated pathways (i.e., “Fatty Acid Biosynthesis”, “Lipid Metabolism Pathway”, “Senescence and Autophagy in Cancer”, and “Focal Adhesion-PI3K-Akt-mTOR-signaling pathway”, Figure S4) were shown to differ between the TNBC and the luminal A subtypes of breast cancer (Figure 7C). Next, results of GSEA [45] revealed significant enrichment (*p* < 0.05) and demonstrated that the TNBC and the luminal A signatures, respectively, were positively and negatively correlated with the ferroptosis gene set, but not other pathways (Figure 7C, Table S3) deposited in the Wikipathways database (Figure 7D). These results corroborate the strong connectivity between ferroptosis and TNBC.

To further characterize prognostic ferroptosis DEGs, univariate COX regression was used to correlate overall survival of the patients with the DEGs (FDR < 0.05, 67 driver and 39 suppressor genes; Figure 7E, the left panel) between TNBC and luminal A that uniquely intersected with either the experimentally validated ferroptosis driver or suppressor genes deposited in the FerrDb (Table S4) [46]. These ferroptosis-related prognostic DEGs (Table S5) were then divided into four clusters (ferroptosis drivers or suppressors in TNBC or luminal A based on expression levels; Figure 7E, right panel). Prognostic ferroptosis driver DEGs (FDR < 0.05) and prognostic ferroptosis suppressor DEGs (FDR < 0.05) were allocated to cluster 1 and 2, and 3 and 4, respectively. Cluster 1 and 3 comprise upregulated genes (log2 (TNBC/luminal A) > 0), and cluster 2 and 4 comprise downregulated genes (log2 (TNBC/luminal A) < 0). Furthermore, ferroptosis-related prognostic DEGs in the clusters (Figure 7E) were adopted to acquire FPI (Table S6) [48] by using GSVA [49] for the calculation of sample-wise gene set enrichment scores. FPI has been utilized to model the propensity of a certain condition (e.g., drug treatment and cancer aggressiveness) to the induction of ferroptosis in multiple cancer types from The Cancer Genome Atlas datasets and cancer cell lines [48]. The observation of higher FPI (*p* < 0.0001) in patients with TNBC compared with luminal A patients (Figure 7F) suggests that there is a higher potential for inducing ferroptosis in TNBC than in luminal A. These results, based on the transcriptomic data of human patient specimens, confirmed that TNBC was more sensitive to ferroptosis than luminal A (Figure 1 and Figure 2).

## 4. Discussion

By screening various natural compounds using ferroptosis inhibitor Fer-1, FC induced ferroptosis more effectively in TNBC MDA-MB-231 cells compared to luminal A MCF-7 cells (Figure 1A). FC, also termed Paris saponin II [55], is a structurally defined [56] diosgenin saponin isolated from *Paris formosana Hayata* (Liliaceae), which has been used as a folk remedy for snakebite inflammation and tumors. Immunological, anti-inflammatory, anti-cancer, and anti-bacterial properties of FC have been reported [55,56,57,58,59,60]. We have previously demonstrated that FC induces apoptosis in human colorectal cancer HT-29 cells via mitochondrion- and caspase 2-related pathways [25]. The apoptotic effect of FC has also been demonstrated in human hepatocellular carcinoma HepG2 cells [60]. In animal studies, the anti-tumor effects of FC on xenografts of human ovarian SKOV3 and colorectal HCT 116 cancer cells have been reported to proceed via the inhibition of NF-κB [55] as well as the fission of mitochondria [61]. Besides apoptosis, FC treatment induces paraptosis and sensitizes lung NCI-H460 and NCI-H520 cancer cells to cisplatin in a manner depending on the JNK pathway, endoplasmic reticulum stress, and mitochondrial swelling [62]. FC also enhances the polyphyllin I-induced cytotoxicity of HepG2 liver cancer cells via cell cycle arrest at the G1 phase and a mitochondrion-dependent apoptotic pathway [59]. Synthetic lethality of FC has also been observed in combination with polyphyllin VII, another *Rhizoma Paridis* saponin, in human lung NCI-H460 cells via activation of caspases and cleavage of Beclin 1 [63]. These results suggest that FC may have chemotherapeutic potential against various types of human cancers via various death mechanisms. The results of Figure 1 indicate that FC-suppressed cell growth was rescued only slightly, although significantly, by Fer-1. This phenomenon is very different from the treatment with erastin or RSL3, in which the rescue by Fer-1 is almost complete. These observations indicate that cell death mechanisms other than ferroptosis may be associated with FC-reduced cell growth. The FC-induced formation of autophagosomes/autolysosomes in MDA-MB-231 cells (Figure 4C) and FC-elevated protein expression of autophagy marker LC3-II/LC3-1 in both MDA-MB-231 cells (Figure 3) and MCF-7 cells (Figure S1) suggest the involvement of autophagy. Furthermore, the FC-reduced full length of caspase 3 and PARP in MDA-MB-231 cells (Figure S5) and PARP in MCF-7 (Figure S1) imply the induction of apoptosis. In FC-induced ferroptosis, FC treatment in MDA-MB-231 cells increased transferrin receptor 1 (data not shown) and decreased ferroportin (Figure 3) protein expressions at 24 h, resulting in an increase in protein level of FTH1 at 24 h. The FC-induced ferritinophagy at 24 h (Figure 4) and FC-induced autophagy at both 24 and 48 h (Figure 3) may accelerate protein degradation of FTH1 and cause no significant elevation of FTH1 at 48 h (Figure 3). The increase in labile iron pool (Figure 2D) possibly elevated ferroportin for iron-efflux and counteracted the FC-reduced ferroportin, leading to no significant changes of ferroportin protein expression at 48 h (Figure 3).

In the present study, we showed that FC and RSL3 triggered ferroptosis to a greater degree in TNBC MDA-MB-231 cells as compared to luminal A MCF-7 cells (Figure 1). The discrepancies between TNBC and luminal A with respect to ferroptosis were corroborated by the gene analysis outcomes using a database of patients (Figure 7). Besides generating ROS through the tricarboxylic acid cycle and electron transport chain, mitochondria are indispensable for ferroptosis induction by either cystine-deprivation or erastin treatment, but not by GPX4 inhibition [64]. Herein, the results of RNA sequencing suggest an association with the electron transport chain in cells exposed to FC (Figure 5B). Furthermore, glutaminolysis, acting through an anaplerosis reaction, is required for both cystine deprivation- and erastin-induced ferroptosis [64]. Glutamic-oxaloacetic transaminase 1, an enzyme converting glutamate to α-ketoglutarate for acetyl-CoA production and subsequent lipid and ROS synthesis [65], and solute carrier family 1 member 5, a protein mediating the uptake of L-glutamine for deprivation of cystine and prevention of glutathione formation [65], are involved in glutamine metabolism and their expressions are greater in TNBC as compared to the luminal A subtype (Figure 7E). Collectively, it is plausible that FC triggers ferroptosis in TNBC MDA-MB-231 cells partially through the activation of the electron transport chain.

The molecular heterogeneity of breast cancer is well recognized. The calculated Nottingham prognostic index from the METABRIC cohort (Figure 7B) indicates that TNBC is more aggressive and has a poor prognosis as compared to luminal A. To effectively facilitate therapeutic strategies and to overcome the variation in the clinical outcomes of patients, the molecular diversity of breast cancer patients’ specimens collected by the METABRIC cohort was applied to test the differences between TNBC and luminal A. Ferroptosis and autophagy were identified to explain the complex genomic landscape that underlies the disease (Figure 7C,D). In light of ferroptosis being an autophagy-related cell death mechanism, the driver and suppressor genes of ferroptosis from FerrDb, the first and comprehensive database of ferroptosis developed in 2020, were integrated with the patients’ overall survival status from the METABRIC cohort to characterize the molecular diversity with prognostic value. Based on the gene expression profiles in TNBC and luminal A, the prognostic ferroptosis DEGs were further stratified into four clusters (Figure 7E). Functional analysis of these prognostic ferroptosis DEGs was conducted and the results demonstrating the high FPI in TNBC provided further evidence that the molecular profile of TNBC is sensitive to ferroptosis (Figure 7F) which is in accordance with the results from The Cancer Genome Atlas breast cancer cohort [48]. Transcriptome-guided therapeutic strategies identify the induction of ferroptosis, probably in an autophagy-related fashion (ferritinophagy), as a promising approach to treat TNBC patients. The 12 ferroptosis driver (cluster 1) and suppressor (cluster 4) genes with increased and decreased expression in TNBC specimens, respectively, may contribute to the sensitivity of TNBC to ferroptosis. Targeting the ferroptosis suppressor genes that are upregulated in TNBC (four genes in cluster 3) and/or raising the ferroptosis driver genes that are downregulated in TNBC (four genes in cluster 2) may enhance ferroptosis in TNBC (Figure 7E, right panel). According to the biological functions of these 20 genes, decreased antioxidant capacity, dysregulated cellular iron homeostasis, and increased synthesis of polyunsaturated fatty acids containing phosphatidylethanolamine, may favor ferroptosis induction in TNBC patients. This notion is in line with the three hallmarks of ferroptosis: the oxidation of polyunsaturated fatty acid-containing phospholipids, redox-active iron, and inhibition of lipid peroxide repair [66].

Most of the 25 selenoproteins in humans have been demonstrated or predicted to perform oxidoreductase activities [67]. Among the five selenium-dependent glutathione peroxidases in humans, cytosolic glutathione peroxidase 1 catalyzes the decomposition of H_2_O_2_ [68], but GPX4 has a distinctive substrate preference toward phospholipid hydroperoxide for the protection of biological membranes [69,70]. It has been recently reported that inhibition of GPX4 activity promotes H_2_O_2_-induced ferroptotic cell death in mice [69]. The observation of prioritized GPX4 expression at the expense of low-hierarchy selenoproteins, such as glutathione peroxidase 1 under selenium deficiency [71,72], suggests that GPX4 plays essential roles in the prevention of membrane oxidation. Likewise, the GPX4 activity inhibitor RSL3 [13] was identified through the approach of synthetic lethal screening from a total of 47,725 compounds [73]. RSL3 binds directly with and inhibits the activity of GPX4 [13,69], resulting in a rapid accumulation of lipid ROS, phospholipid oxidation, and ferroptosis [6]. In the present study, we suggest FC as a novel GPX4 regulator by repressing the expression of GPX4 (Figure 2C).

The platinum complex cisplatin has been used to treat a number of cancers for decades. Although cisplatin has a couple of side effects, such as nephrotoxicity [74], it is still a mainstay in the therapy of solid tumors, including breast cancers [75]. Recent studies have found that cisplatin acts as an inducer of ferroptosis in human non-small cell lung cancer A549 and colorectal carcinoma HCT116 cells through glutathione depletion and inactivation of glutathione peroxidase activities [76], despite demonstrating weaker efficacy in comparison to another ferroptosis inducer, erastin. Our data indicate that FC-induced cell growth inhibition was greater than that induced by cisplatin in TNBC MDA-MB-231 cells, but not in luminal A MCF-7 cells (Figure 1A). In fact, cisplatin-induced lipid ROS formation (Figure 6B) slightly but significantly causes cell growth inhibition in MDA-MB-231 cells (Figure 6A), and the cell growth inhibition was significantly reversed by co-administration of Fer-1 (Figure 1A), suggesting that cisplatin alone significantly contributes to ferroptosis. Enhancement of cisplatin-induced cell growth inhibition and ferroptosis by FC was also evidenced in MDA-MB-231 cells (Figure S3A), which is in line with the gene analysis results, in which the enriched pathways were analyzed via CPDB (Figure 5B). Importantly, the 50% inhibitory concentrations for FC in normal human peripheral blood mononuclear cells and umbilical vein endothelial cells are at least 10 and 20 times, respectively, and higher than those in human colorectal cancer HT-29 and human hepatocellular carcinoma Hep3B cells [25]. Thus, FC is relatively safe in human normal cells as compared to human cancer cell lines, suggesting that dietary phytochemicals recovered from herbs and spices are promising for drug discovery.

In terms of its biological behavior, TNBC is usually more aggressive, more prone to early recurrences, and more likely to be detected in distant metastases as compared with other breast cancer subtypes. Our results provided novel insights and demonstrated that, in comparison to the luminal A MCF-7 cells, RSL3-induced ferroptosis was more prominent in TNBC MDA-MB-231 cells. Saponin FC was more effective at activating ferroptosis in MDA-MB-231 cells, as evidenced by enhanced iron metabolism, oxidative stress, and ferritinophagy. The enhanced cell growth inhibition by FC in cisplatin-treated MDA-MB-231 cells suggests the therapeutic potential of FC in TNBC by induction of ferroptosis. Using transcriptomic data from primary specimens of breast cancer patients in the METABRIC cohort, we confirmed that TNBC was more sensitive than luminal A breast cancer to ferroptotic cell death. We acknowledge that selecting DEGs based on both significance (e.g., *p*-value) and fold change is more robust than either alone. However, the objective of the present study is evaluating the involvement of ferroptosis in the action of FC (Figure 5) and discrepancy between patients with TNBC and luminal A subtypes (Figure 7), but not identifying specific genes modulating ferroptosis. Therefore, to include more genes for further analysis (i.e., overrepresentation analysis), we defined DEGs here as genes with *p*-value < 0.05 (Figure 5) or FDR < 0.05 (Figure 7) without fold change thresholding. Our data demonstrate that induction of ferroptosis could be an important therapeutic tool in the management of TNBC.

## 5. Conclusions

This study identified ferroptosis as a targetable metabolic niche in TNBC via integrated bioinformatics analysis, and these results open up a new avenue of research aimed at treating this aggressive cancer. Our findings shed new light on the mechanisms by which FC, a natural saponin, induces ferroptosis and ferritinophagy, and increases the chemosensitivity of TNBC MDA-MB-231 cells to cisplatin.

## Data Availability

Data are contained within the article and Supplementary Materials.

## Supplementary Materials

The following supporting information can be downloaded at: https://www.mdpi.com/article/10.3390/antiox11020298/s1, Table S1: The DEGs between FC treatment and vehicle. [Method 2.9]; Table S2: The over-represented pathways between MDA-MB-231 cells exposed to either FC or vehicle. [Method 2.11], Table S3: The over-represented pathways between patients with TNBC subtype and luminal A subtype in METABRIC. [Method 2.11], Table S4: Experimentally validated and unambiguous ferroptosis driver genes and suppressor genes in FerrDb. [Method 2.12], Table S5: Ferroptosis DEGs between patients with TNBC subtype and luminal A subtype in METABRIC. [Method 2.12], Table S6: Enrichment scores of prognostic ferroptosis DEG sets in patients with TNBC subtype and luminal A subtype in METABRIC and their corresponding FPIs. [Method 2.13], Figure S1: Effect of FC on the expressions of proteins related to antioxidant system, iron metabolism, and apoptosis in MCF-7 cells. Figure S2: Interaction of LC3 and FTH1 demonstrated by immunoprecipitation. Figure S3: Ferroptosis inhibitor reversed FC-enhanced biosensitivity and lipid ROS formation in cisplatin-treated cells. Figure S4: GSEA of fatty acid biosynthesis, lipid metabolism pathways, senescence and autophagy in cancer, and focal adhesion-PI3K-Akt-mTOR-signaling pathway gene set against gene expression of TNBC and luminal A subtypes. Figure S5: FC changes expressions of apoptosis-related proteins in MDA-MB-231 cells.

## Abbreviations

CPDB, Consensus Path Database; DEGs, differentially expressed genes; ER, estrogen receptor; FC, formosanin C; FDR, false discovery rate; Fer-1, ferrostatin-1; FPI, ferroptosis potential index; FTH1, ferritin heavy chain 1; GPX4, glutathione peroxidase 4; GSEA, gene set enrichment analysis; GSVA, gene set variation analysis; H2DCFDA, 2′,7′-dichlorodihydrofluorescein diacetate; HER2, human epidermal growth factor receptor 2; LC3B, microtubule-associated proteins 1A/1B light chain 3B; METABRIC, Molecular Taxonomy of Breast Cancer International Consortium; NCOA4, nuclear receptor co-activator 4; PR, progesterone receptor; ROS, reactive oxygen species; RSL3, 1S,3R-Ras-selective lethal small molecule 3; SEMs, standard errors of the means; TNBC, triple-negative breast cancer.
