# Supplementary material for: Vulnerability of Triple-Negative Breast Cancer to Saponin Formosanin C-Induced Ferroptosis
Source: Antioxidants (Basel). 2022 Jan 31;11(2):298. doi: 10.3390/antiox11020298 (PMC8868405; doi:10.3390/antiox11020298)
Supplement: Supplementary file 1 [file antioxidants-11-00298-s001.zip › Supplementary Materials/Supplementary data.pdf]

Supplementary Materials

Table S1. The DEGs between FC treatment and vehicle. [**Method 2.9**]

Table S2. The over-represented pathways between MDA-MB-231 cells exposed to either FC or vehicle. [**Method 2.11**]

Table S3. The over-represented pathways between patients with TNBC subtype and luminal A subtype in METABRIC. [**Method 2.11**]

Table S4. Experimentally validated and unambiguous ferroptosis driver genes and suppressor genes in FerrDb. [**Method 2.12**]

Table S5. Ferroptosis DEGs between patients with TNBC subtype and luminal A subtype in METABRIC. [**Method 2.12**]

Table S6. Enrichment scores of prognostic ferroptosis DEG sets in patients with TNBC subtype and luminal A subtypes in METABRIC and their corresponding FPIs. [**Method 2.13**]

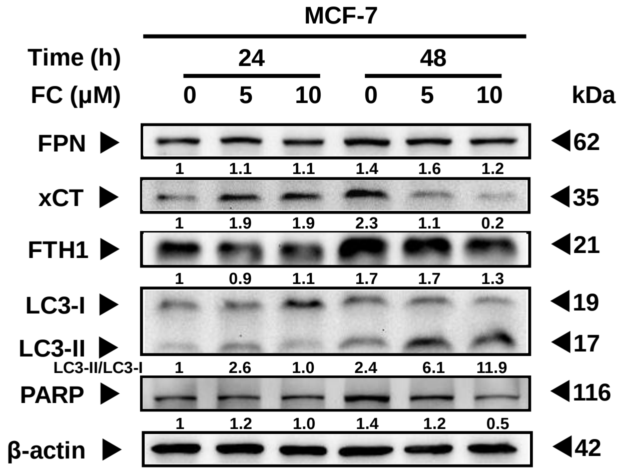

Figure S1. Effect of FC on the expressions of proteins related to antioxidant system, iron metabolism, and apoptosis in MCF-7 cells. Cells received treatments of FC for 24 and 48 h. Whole cell lysates were prepared and subjected to Western blot analysis using anti-ferroportin, anti-xCT, anti-FTH1, anti-LC3, and anti-PARP antibodies. β-actin antibody was used as an internal control. The intensity of each protein expression band was quantified and normalized to the expression of β-actin, with the control level arbitrarily set to 1. FPN denotes ferroportin.

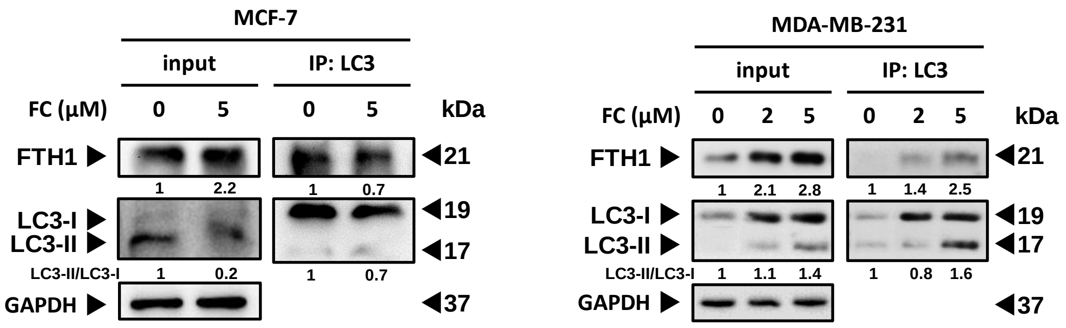

Figure S2. Interaction of LC3 and FTH1 demonstrated by immunoprecipitation. After treatment for 24 h, the cell lysates of MDA-MB-231 immunoprecipitated with anti-LC3 antibody were subjected to Western blot using anti-LC3 and anti-FTH1 antibody separately as the description in the Material and Methods. After treatment for 24 h, MCF-7 cells were harvested in a lysis buffer. The cellular protein (1 mg) was incubated with LC3 antibody at 4°C overnight. Protein G agarose beads (60 μl) (GE Healthcare, UK, 17-0618-01) were incubated with the immuno-complexes for 1 h at room temperature. The beads containing proteins and antibody were collected after centrifugation. With the use of RIPA buffer, the protein expression of FTH1, LC3, and GAPDH (internal control) was detected by immunoblotting. The intensity of each protein expression band was quantified and normalized to the expression of GAPDH, with the control level arbitrarily set to 1.

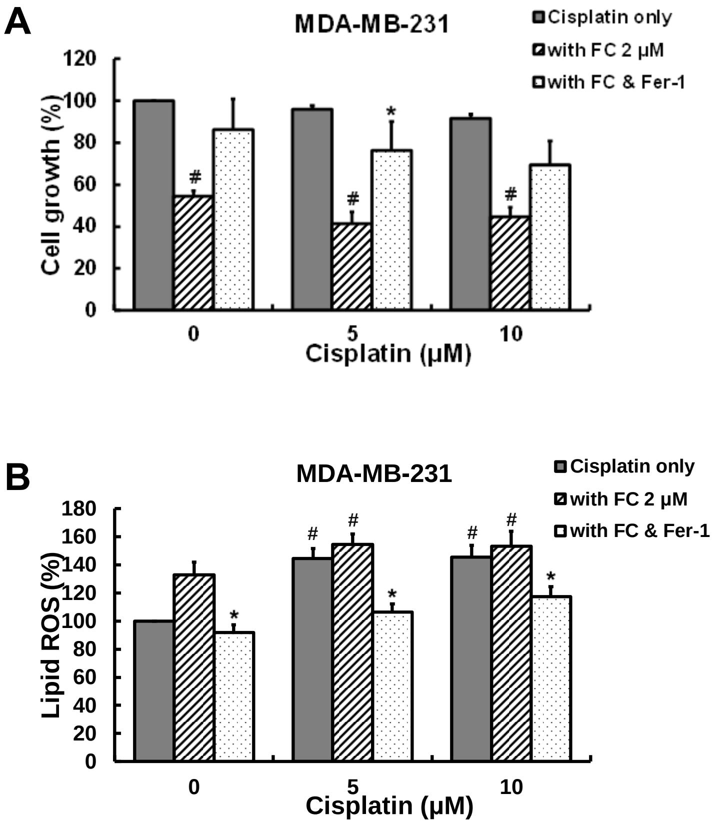

Figure S3. Ferroptosis inhibitor reversed FC-enhanced biosensitivity (A) and lipid ROS formation (B) in cisplatin-treated cells. Cells received treatments of cisplatin in the presence and absence of FC or FC in combined with ferroptosis inhibitor ferrostatin-1 (Fer-1, 5 μM) for 24 h. Cell growth was analyzed by sulforhodamine B assay. Lipid ROS were detected using flow cytometry after staining with C11-BODIPY. * Compared to with FC 2 μM, $p < 0.05$; Student's t-test. # Compared to cisplatin only (A) or compared to the corresponding vehicle (B), $p < 0.05$; Student's t-test.

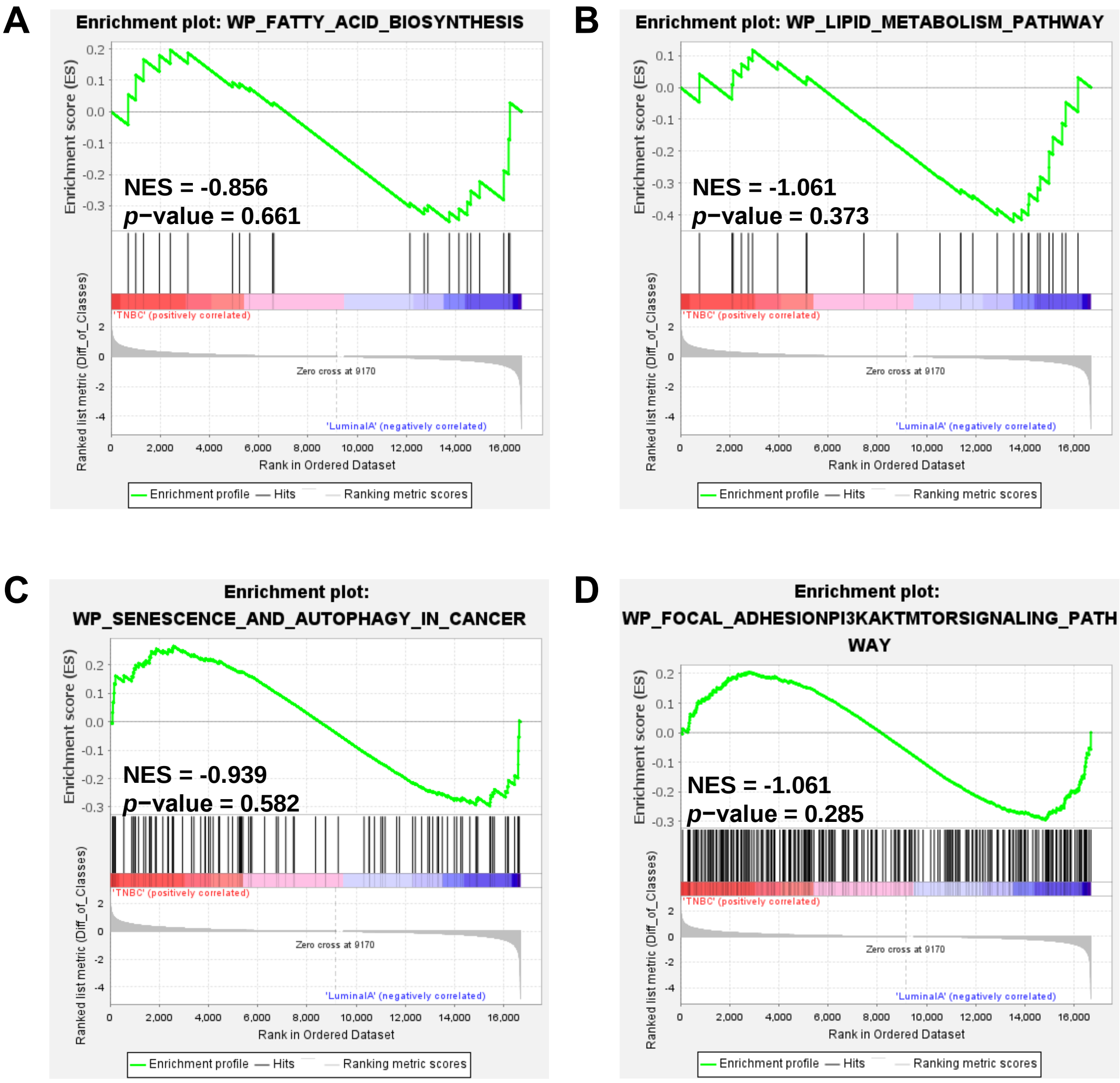

Figure S4. GSEA of (A) Fatty acid biosynthesis, (B) Lipid metabolism pathways, (C) Senescence and autophagy in cancer, and (D) Focal adhesion-PI3K-Akt-mTOR-signaling pathway gene set against gene expression of TNBC and luminal A subtypes.

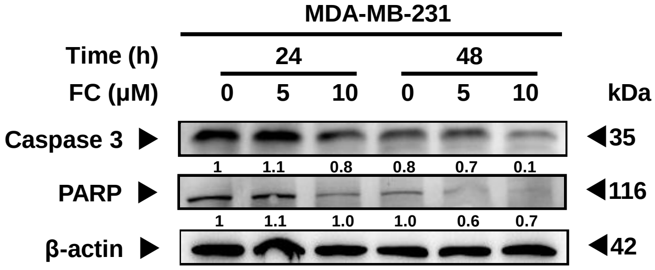

Figure S5. FC changes expressions of apoptosis-related proteins in MDA-MB-231 cells. Cells received treatments of FC for 24 and 48 h. Whole cell lysates were prepared and subjected to Western blot analysis using anti-caspase 3 and anti-PARP antibodies. β-actin antibody was used as an internal control. The intensity of each protein expression band was quantified and normalized to the expression of β-actin, with the control level arbitrarily set to 1.
